# Stealing nephrons—a review on how patent ductus arteriosus physiology impacts neonatal kidney health

**DOI:** 10.1038/s41372-025-02477-w

**Published:** 2025-11-05

**Authors:** Paige E. Condit, Lyndsay A. Harshman, Danielle E. Soranno, Adrianne R. Bischoff, Matthew W. Harer, Patrick J. McNamara

**Affiliations:** 1 Division of Neonatology, Department of Pediatrics, University of Wisconsin School of Medicine and Public Health, Madison, WI USA; 2 Division of Nephrology, Dialysis and Transplantation, Department of Pediatrics, University of Iowa Carver College of Medicine, Iowa City, IA USA; 3 Division of Nephrology, Department of Pediatrics, Indiana University, Riley Hospital for Children, Indianapolis, IN USA; 4 Division of Neonatology, Department of Pediatrics, University of Iowa Carver College of Medicine, Iowa City, IA USA

**Keywords:** Pathogenesis, Diagnostic markers

## Abstract

Adverse short- and long-term kidney outcomes are increasingly recognized as sequelae of NICU care, oftentimes of multifactorial origin and in the setting of a patent ductus arteriosus (PDA). Many studies have reviewed the interactions between the PDA and the brain, intestine, and lungs, but few have specifically reviewed the potential influence of the PDA on the kidney. This review describes the current breadth of literature as it relates to the pathophysiologic interplay between the PDA and the kidneys, as well as how PDA intervention may influence kidney health while neonates are still in the NICU and after discharge. We also discuss novel methods of monitoring these interactions with biomarkers and near infrared spectroscopy, and highlight areas with a dearth of evidence to promote future study.

## Introduction

Infants born preterm (<37 weeks’ gestation) and especially those born at very low birth weights (VLBW) < 1500 g are at increased risk of short- and long-term kidney morbidities. The incidence of acute kidney injury (AKI) among neonates admitted to the NICU is reported as between 18%-70%, with a higher incidence proportional to lower gestational age [1–3]. Further, premature infants are at increased risk of developing chronic kidney disease (CKD) later in life [4–7]. Several neonatal exposures and consequences increase the risk of AKI, including reduced nephron endowment, nephrotoxic medication exposures, and pathological conditions such as sepsis, fluid overload, and intra- and extracardiac shunts. The patent ductus arteriosus (PDA) is the most common hemodynamic problem of prematurity, with rates between 33-66% in VLBW neonates [8, 9]. Of note, moderate to high volume PDA shunts may lead to kidney hypoperfusion and contribute to AKI, with rates of AKI reportedly occurring in 30–50% of neonates with a PDA [10–12]. Current methods for PDA management include fluid restriction and use of medications, including nonsteroidal anti-inflammatory drugs (NSAIDs), acetaminophen, and diuretics, as well as procedural closure with either surgical ligation or transcatheter closure. Yet each of these treatment methods has the potential to negatively impact kidney function. These effects may be cumulative after exposure to fluid restriction and NSAIDs, but as the shunt remains, it continues to independently compromise renal perfusion.

Given the prevalence of a persistent PDA in VLBW infants and its impact on kidney perfusion, it is important to consider the role of a PDA in both short- and long-term kidney outcomes. Recent neonatal nephrology reviews have highlighted kidney health as a growing area of focus, underscoring the need for deeper investigation into how a PDA may contribute to adverse kidney sequelae. This review summarizes the current literature on the relationship between PDA and kidney outcomes, with an emphasis on opportunities for future research. In contrast to previous reviews, we present new data related to the effects of shunt volume on nephron injury, biomarkers for kidney injury prevention, and the impact of PDA effects on longer term kidney health.

## Pathophysiology

### Proposed mechanism

The persistence of a patent ductus arteriosus (PDA) in premature infants can be attributed to several factors. In utero, ductal patency is maintained by elevated levels of prostaglandins and nitric oxide, as well as low oxygen tension—conditions that undergo significant changes after birth. Postnatally, the oxygen-driven upregulation of calcium channels is diminished in preterm infants, resulting in reduced calcium influx and impaired smooth muscle constriction, which limits functional closure of the ductus [13]. Animal studies have further demonstrated that the preterm ductus is more sensitive to vasodilators such as prostaglandins and nitric oxide, reinforcing its tendency to remain open [14, 15]. Additionally, infection-induced prostaglandin production, particularly in the context of sepsis, may prevent closure or even cause reopening of the PDA [16]. Pathology related to the PDA may occur when there is failure of normal postnatal closure and there are hemodynamic consequences to pulmonary (i.e., over circulation) and systemic blood flow (i.e., hypoperfusion).

**Fig. 1:**
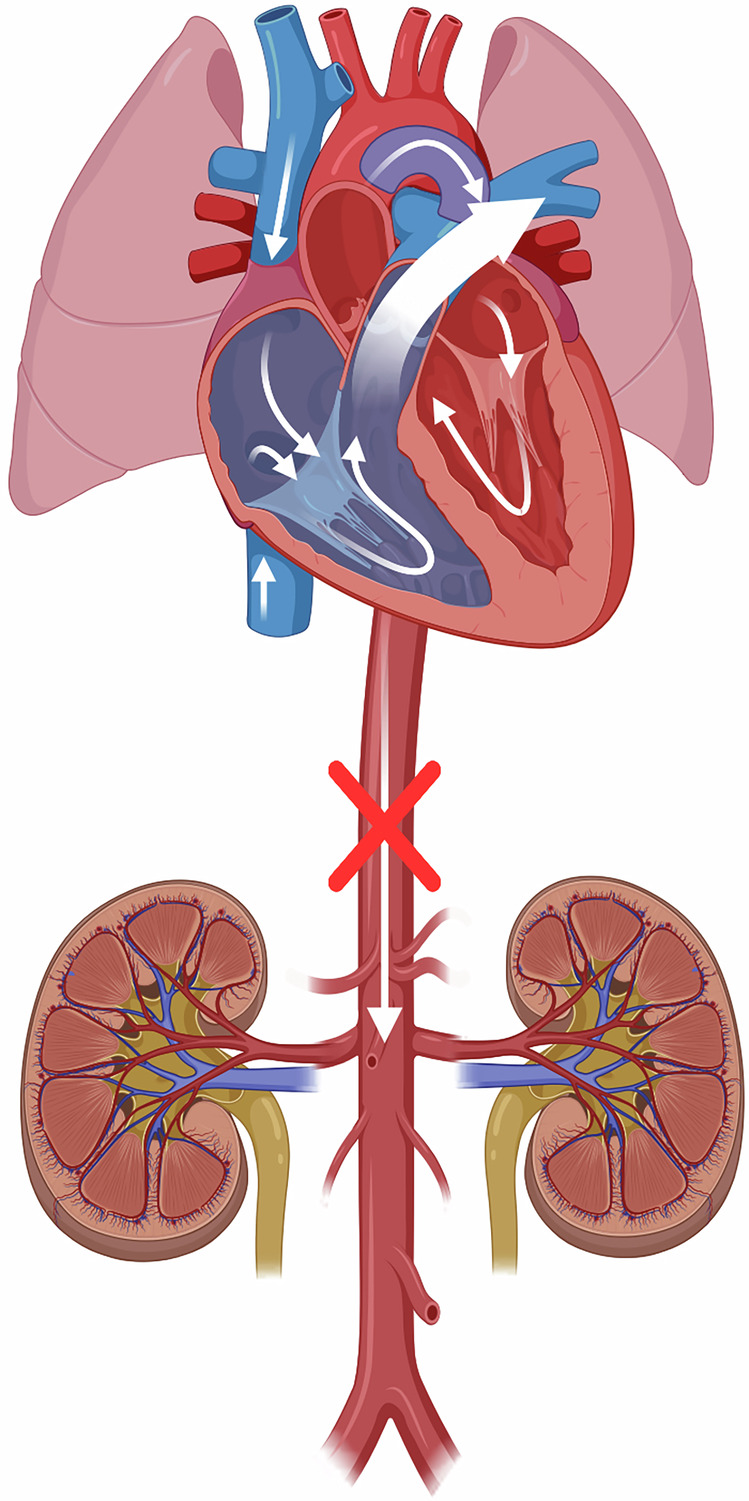
Representation of ductal steal with increased blood flow to the lungs and decreased blood flow distally in the aorta; created in BioRender. Harer, M. (2025) https://BioRender.com/f02w6p6.

As systemic vascular resistance increases, the transductal pressure gradient changes such that the amount of left-to-right shunt increases, thereby leading to increased pulmonary blood flow and decreased post-ductal perfusion. This concept of lower post-ductal blood flow is oftentimes referred to as ‘ductal steal’ and, depending on PDA shunt volume, has been associated with increased risk of conditions like necrotizing enterocolitis (NEC) and AKI [12, 17, 18] (schematically represented in Fig. 1). In this setting, the PDA is frequently referred to as ‘hemodynamically significant’; however, the exact definition of hemodynamically significant is widely debated and inconsistent in clinical trials of PDA treatment [19].

The hemodynamic consequences of a pathologic shunt relate to the burden (magnitude and duration) of the PDA shunt; therefore, understanding of the biologic determinants of transductal flow is essential for clinicians. The Hagen-Poiseuille equation $(Q=\,\frac{\pi P{r}^{4}}{8\eta l})$ where volume of flow (Q) is proportional to the pressure difference across a cylinder (P) and the radius (r) to the fourth power and inversely proportional to the viscosity of the liquid (η) and the length of the tube (l), forms the basis for understanding the conditions which may maintain ductal patency and increased flow across the ductus. Conditions that promote decreases in pulmonary vascular resistance and increased transductal gradient, such as hyperoxia, hypocapnia, inhaled nitric oxide use, or administration of surfactant, lead to increased flow and higher shunt volume. Anemia leading to decreased viscosity, increased PDA diameter, and decreased PDA length may all contribute to increased transductal flow and failure of normal postnatal closure.

#### Pathological effects on the kidneys

Neonatal kidneys are vulnerable to the effects of hypoperfusion due to their differences in structure and function from their term counterparts. Nephrogenesis is completed between weeks 34-36 of gestation, with term infants being born with a full complement of nephrons; however, functionally, kidneys continue to develop in the neonatal period and infancy [20]. While the kidneys continue to grow in preterm neonates, nephron number only increases for a limited period postnatally until a final nephron number is achieved, leaving preterm infants reduced nephron endowment [21–23]. This reduction in functioning nephrons portends an increased workload per nephron, predisposing to glomerular hyperfiltration with a resultant risk of glomerulosclerosis. Decreased blood flow from increased ductal shunting may further stress these nephrons.

Renal artery blood flow (RBF) in fetal life accounts for 3–7% of the combined cardiac output. Following the rise in systemic vascular resistance, decrease in renal vascular resistance, and increased cardiac output that occurs after birth, RBF increases to 10% of cardiac output in the first week of life and eventually to the typical 25% of the cardiac output seen in adulthood by 2 years of age [24]. Multiple studies have shown the presence of a PDA—defined in these studies as those with diastolic flow reversal—decreases RBF and that improved RBF occurs after ductal closure [25, 26]. Therefore, understanding the relationship of moderate-high volume PDA shunts and kidney injury is a high priority for clinicians.

## PDA echocardiographic diagnosis

Various echocardiography parameters can be used to characterize PDA shunt volume [27]. Unfortunately, most studies have used a single point non-standardized estimate of ductal diameter in isolation to define hemodynamic significance [28–31]. Of note, this definition has not been shown to correlate well with adverse outcomes [32]. In addition, most clinical trials of PDA treatment have randomized babies based on PDA diameter alone. It is, therefore, not surprising that intervention was not associated with improved neonatal outcomes. PDA diameter is a poor surrogate of true shunt volume because there is a wide range of sizes of neonates, and the diameter is relative; a 3 mm shunt in a 3500 g neonate compared to an 800-gram neonate is different [33].

Other composite PDA scores have been developed more recently that include multiple echocardiography measures, including the ductal diameter (indexed to the patient’s weight) as well as measurements of left heart volume overload (i.e., left atrium to aorta root ratio and left ventricular output to right ventricular output ratio) and systemic hypoperfusion. The relationship of PDA composite scores and adverse neonatal outcomes is stronger than ductal diameter alone [34, 35]. Table 1 shows two composite echocardiography PDA scores used to determine hemodynamic significance. These composite scores include multiple parameters that are indicative of shunt volume and not simply physical size alone.

**Table 1:** Components of the Iowa PDA score and El-Khuffash PDA severity score both used to determine hemodynamic significance.

Iowa PDA Score	El-Khuffash PDA Severity Score
Pulmonary vein D wave (cm/s)	Gestation (weeks)
Mitral valve E wave (cm/s)	PDA diameter (mm)
Isovolumetric relaxation time (ms)	Left ventricular output (mL/kg/min)
Left atrium to aortic root ratio	Maximum PDA velocity (m/s)
Left to right ventricular output ratio	Left ventricle a’ wave (cm/s)
Aortic/Peripheral Doppler flow reversal	
Ductus diameter indexed to weight (mm/kg)	

## Acute kidney injury

### AKI diagnosis

The neonatal modified Kidney Disease Improving Global Outcomes (KDIGO) definition [36] is the consensus definition of neonatal AKI developed in 2013 at a multidisciplinary National Institutes of Health workshop devoted to neonatal AKI. This definition stages AKI into three groups, with stage three being the most severe (Table 2) [36, 37], and allows for the diagnosis of neonatal AKI based on either changes in serum creatinine or urine output. While this is the gold standard for clinical and research-based AKI diagnosis, several challenges remain with this definition, including the accuracy of diagnosis of AKI in the NICU. For example, challenges persist in obtaining timely collection and measurement of both serum creatinine and urine output. Defining a baseline serum creatinine to compare levels is inherently problematic in neonates. In the early postnatal weeks serum creatinine should be physiologically declining as GFR is improving, and as the initial serum is in isoequilibrium with maternal levels (i.e., at birth serum creatinine reflects maternal kidney function) [38, 39]. Further, in the NICU, where blood volume and lab draws are scrutinized, having regular measurements to detect changes in a timely manner is not realistic [40]. Lastly, changes in creatinine representative of AKI lag behind the etiologic event. Furthermore, it is critical to note that serum creatinine does not noticeably increase until a 50% decrease in the glomerular function has already occurred—impeding the utility in timely intervention [41, 42]. Collection of accurate urine output is also challenging in the NICU as indwelling catheters are associated with increased infection risks, diaper weights are inaccurate, and there is frequent stool contamination. These challenges often lead to the exclusion of urine output criteria in retrospective research evaluating neonatal AKI [43].

**Table 2:** Neonatal Modified KDIGO criteria for AKI.

Stage	Serum Creatinine	Urine Output
1	1.5–1.9 times baseline ≥ 0.3 mg/dL increase (48 h)	<0.5 mL/kg/hr for 6 h
2	2.0–2.9 times baseline	<0.5 mL/kg/hr for 12 h
3	3 times baseline ≥ 2.5 mg/dL increase	<0.3 mL/kg/hr for 24 h Anuria for 12 h

### AKI epidemiology

The AWAKEN study evaluated the overall incidence of AKI in the NICU and found that the incidence of AKI in a world-wide cohort was 30%. After controlling for pertinent confounders, including gestational age and birth weight, AKI was associated with a 7.5 increased odds ratio (4.5–12.7) of mortality and a 14.9 (11.6–18.1) day increased length of stay [1]. The incidence of AKI was also shown to vary by gestational age, with infants born between 22 to less than 29 weeks’ gestation having the highest incidence of AKI (47.9%), those born between ≥29 to less than 36 weeks’ gestation having an incidence of 18.3%, and those born ≥ 36 weeks’ gestation having an incidence of 36.7%.

### Kidney effects of PDA management

PDA management options include expectant management with fluid restriction and diuretic therapy, pharmacologic management with NSAIDs and/or acetaminophen, or interventional PDA closure (transthoracic surgery or transcatheter device closure), all of which could independently be detrimental to kidney health and increase risk of AKI. Fluid restriction and diuretics are used to increase blood viscosity, decrease the volume of flow across the ductus, and treat pulmonary edema. Management strategies that restrict fluid or promote diuresis have conflicting evidence as to whether there is effectiveness leading to ductal closure. Although fluid restriction in infants with PDAs has been shown to reduce blood flow in the superior vena cava and superior mesenteric artery, it does not appear to influence PDA closure rates. Notably, while renal arterial blood flow was not directly assessed, it is reasonable to hypothesize that it may also be compromised, given that the effects of fluid restriction are not confined to the pulmonary vascular bed or the PDA itself [44]. Diuretic therapy, in particular furosemide, has not been shown to promote ductal closure and may actually increase ductal patency through renal production of prostaglandins [45]. Furosemide has also been associated with adverse kidney and neonatal outcomes in the NICU [46, 47]. Medications used to induce ductal closure include NSAIDs—ibuprofen and indomethacin—or acetaminophen. Both ibuprofen and indomethacin have been shown to also have negative renal effects through decreasing synthesis of prostaglandins, which impairs vasodilation of the afferent arteriole leading to decreased renal blood flow and GFR, resulting in oliguria and renal insufficiency [48–50]. Given that many of these pharmacologic treatments are used together, there could be a multiplicative effect on kidney health. If medical treatment strategies fail to achieve PDA closure or resolution of hemodynamic significance, the negative kidney effects are added to the effect of hypoperfusion caused by the PDA and can serve as a mechanism for more deleterious kidney injury. Interventional PDA closure subjects the neonate to abrupt hemodynamic changes which may lead to post ligation (or catheter occlusion) cardiac syndrome. This condition involves hypotension and decreased cardiac output due to afterload increases and decreased left ventricular function following PDA closure. This can in turn decrease systemic perfusion systemic and increase potential for AKI. It been demonstrated in the literature that infants undergoing interventional PDA closure have increased rates of AKI though in current reports confounders exist including gestational age, birthweight, and nephrotoxic medication exposure that determining the true risk is not yet certain [51, 52].

### AKI in PDA trials

In a sub-analysis of the AWAKEN data, VLBW infants with a PDA, defined as the presence of the diagnosis on the AWAKEN discharge data form, had a 3.7 times increased risk of AKI using the KDIGO definition than their counterparts without a PDA [12]. Physiologically, the higher rates of AKI in the setting of a hemodynamically significant PDA are biologically plausible secondary to post-ductal hypoperfusion as demonstrated in numerous other studies [11]. Other mechanisms of PDA-related AKI include acute-onset post-ductal hypotension leading to decreased renal perfusion pressure [11]. More recently, Wildes et al. found that a high-risk El-Khuffash PDA score, which is a composite measure of various echocardiography parameters to evaluate hemodynamic significance, was associated with an increased risk of AKI [53]. There are, however, limited data of the impact of PDA shunt burden (severity and duration of exposure) on rates of AKI and AKI staging. AKI has not historically been included in many randomized control trials (RCTs) evaluating the PDA and outcomes of PDA treatments. Of the larger RCTs published in the past 10 years, two have included kidney-related outcomes in their secondary outcomes but have not included specific AKI data. The Baby-OSCAR trial conducted by Gupta et al. evaluated whether treatment with ibuprofen within the first 72 postnatal hours for neonates with a PDA diameter greater than or equal to 1.5 mm decreased rates of death or severe bronchopulmonary dysplasia(BPD) [28]. In this trial, infants treated with ibuprofen had a 9.7% increased rate of impaired renal function—defined as a urine output of <0.5 mL/kg/hour and or serum creatinine >100 μmol/L (1.13 mg/dL). The BeNeDuctus Trial conducted by Hundscheid et al. evaluated if treatment with ibuprofen between 24 and 72 hours of age of neonates with a PDA diameter greater than or equal to 1.5 mm and evidence of left to right shunting led to decreased rates of necrotizing enterocolitis, moderate or severe BPD, and or death [29]. Rates of renal failure, defined by a serum creatinine > 120 μmol/L (1.36 mg/dL) or urine output <0.5 mL/kg/hour, were not different between the groups receiving ibuprofen and those with expectant management. Of note, the adjudication of hemodynamic significance was limited so it is difficult to tease out whether there may be independent effects on kidney health according to shunt volume and treatment efficacy. These trials do not demonstrate clear evidence of either kidney protection or harm from PDA treatment; of note, given these measures were secondary outcomes with many confounders present, the associations should be interpreted with caution.

More retrospective studies have evaluated the association between the PDA and effects on the kidney but have increased limitations in their applicability due to potential biases and lack of control over data collection. In one such example, Guillet et al. showed increased incidence of AKI independent of PDA management strategy which may indicate a greater influence of PDA physiology on AKI rates over PDA treatment [12]. Yet, Majed et al. found that in patients treated with ibuprofen or indomethacin, there was a decreased risk of stage 2-3 AKI [11]. Other studies have shown conflicting results that indomethacin can either increase or decrease rates of AKI and renal insufficiency in this population, leaving much to be further evaluated in a more scientifically rigorous fashion [54, 55]. These studies highlight the importance of comprehensive evaluation of hemodynamic significance, classification of patients according to shunt volume and duration of exposure, and independently evaluating the impact of treatment efficacy.

## Novel biomarkers and technology to detect kidney dysfunction and PDA significance

Acute changes in kidney function are traditionally evaluated by serum creatinine and urine output but as stated these markers are not optimal in the neonatal setting. Long-term, kidney dysfunction can be detected using blood-based measures of eGFR in combination with assessment of proteinuria - measured as the spot (random) urine protein to creatinine ratio, radiographic evidence of renal hypoplasia (defined as kidney size less than the 5th percentile bilaterally), and/or the development of hypertension [56]. However, given the limitations of these measures, particularly those used to detect acute changes, work has been done to evaluate other potential biomarkers that may detect changes sooner and more accurately.

Neutrophil gelatinase-associated lipocalin (NGAL) is a secretory protein produced within activated neutrophils and renal tubular epithelial cells. NGAL can be measured in both the serum and urine [57]. Gestational age specific baseline values for urinary NGAL (uNGAL) have been established in neonates to allow for noninvasive measurement making it a more amenable detection tool in the NICU [58, 59]. In general, uNGAL levels decrease with gestational age and there are cutoff values which indicate the presence of acute tubular injury. Further, in a multicenter pooled analysis of both children and adult patients, uNGAL levels increase within hours of injury, 24–72 h before an increase in creatinine [60, 61]. In the premature infant population, uNGAL has been validated to detect AKI in ischemic kidney injury related to exposure to nephrotoxic medications and asphyxia [62, 63]. Given that kidney injury related to a hemodynamically significant PDA is likely to lead to hypoperfusion, this makes uNGAL a potential option for early detection of PDA-associated AKI. A study by Tosse et al. showed that infants who received intervention for their PDA had significantly higher levels of uNGAL prior to their PDA treatment, showing there is likely association between PDA-related ischemia and uNGAL release [64]. Future studies are needed, however, to evaluate the relationship between uNGAL and PDA-related AKI, potentially PDA-related CKD, and PDA treatment.

Kidney injury molecule-1 (KIM-1) is another protein found in the proximal tubule cells and has been shown to be elevated when measured in the urine of premature infants with AKI. There have been fewer convincing studies, however, demonstrating uKIM-1’s utility in isolation and that it is more sensitive when used in combination with other urinary biomarkers for the detection of AKI [65, 66]. Cystatin C is another urinary protein being studied for its potential diagnostic ability in neonatal AKI. It is a protein produced by nearly all cells in the body and is freely filtered by the kidney. Elevated urinary cystatin C levels have been associated with AKI in preterm infants, and trends in association with the PDA have started to be evaluated in recent studies [67, 68]. Capelli et al. found in a small cohort study that levels of cystatin C were higher at specific time points in neonates with a PDA; however, that finding did not persist when multivariable analysis was completed [68].

Urinary metabolomics is an emerging field for diagnosing kidney dysfunction. This differs from the urinary proteomic biomarker detection as the metabolites measured are more reflective of upregulated or downregulated cellular processes related to potential stress instead of the breakdown of cells. Urinary metabolomics have demonstrated differences in profiles in neonates with and without AKI [69]. A study by Bardanzellu et al. found differences in the urinary metabolomic profiles of preterm neonates measured in the first 12 postnatal hours between patients with vs without a hemodynamically significant PDA detected between 48 to 72 h postnatally [70]. Given the relationships demonstrated in both the detection of AKI and hemodynamically significant PDAs, this is a possible tool to allow for improved detection of PDA related effects on kidney health. Currently, use of this tool is limited as it has not been vetted by the FDA and is not readily available within hospital laboratories but shows promise for future studies and use.

Another potential tool to detect perfusion and oxygenation differences within the kidney secondary to a PDA is renal near-infrared spectroscopy (NIRS). NIRS measures tissue oxygenation noninvasively and can be used to calculate fractional tissue oxygen extraction (FTOE) [71]. Several studies have evaluated renal NIRS to detect either the current presence of a hemodynamically significant PDA or to predict the likelihood of developing a PDA requiring treatment, with good sensitivity and specificity. Chock et al. found that a renal regional saturation less than 66% identified a hemodynamically significant PDA with a sensitivity of 81% and specificity of 77%. [72, 73] Similarly, Underwood et al. found that using renal NIRS to screen for PDAs that would require intervention, and therefore could benefit from early echocardiogram evaluation, had a sensitivity of 85% and specificity of 83% [72, 73].

## Chronic kidney disease after PDA

The impact of PDA itself and the various PDA management strategies on long-term kidney function remains unclear. Neonates born preterm (<28 weeks’) are at 2–4 times increased risk of developing CKD, with signs typically developing as early as two years of age [4, 6]. This is felt to be multifactorial in etiology, with the same risk factors that redispose to AKI also predisposing to CKD [21, 22, 74–76]. To our knowledge, only one recent study has specifically evaluated how a PDA may influence longer term kidney outcomes [77]. In this study, investigators demonstrated that infants who received PDA treatment compared to those who did not have no difference in eGFR or albumin to creatine ratio at 2 years of age but did have lower rates of hypertension, defined by systolic blood pressure >90th percentile for age, compared to neonates who did not receive PDA treatment. This may suggest that successful PDA treatment has kidney protective effects. This study is limited by its retrospective nature and lack of comprehensive echocardiography data parameters to adjudicate PDA shunt severity as well as lack of standardized PDA treatment regimens between the comparison groups. There have not been studies completed looking longer term at adult consequences of PDA on kidney health.

## Conclusion

There is a strong physiologic potential explanation for the PDA to affect both the short- and long-term kidney health for preterm infants. Although the relationship between the PDA and development of AKI has been studied, there is limited data on the relationship to shunt burden and treatment efficacy which relates to the lack of standardization of the definition of hemodynamic significance in those studies. There is a unique opportunity to investigate how a hemodynamically significant (moderate to high volume) PDA influences long-term kidney outcomes, including the potential AKI to CKD transition.

Additionally, the use of biomarkers and noninvasive detection tools to monitor kidney health may allow for a more nuanced understand of the physiologic relationship. related to kidney perfusion changes and injury while exposed to a hemodynamically significant PDA. Given what is known about the use of biomarkers and NIRS to monitor kidney changes, there is ample opportunity to study how these tools may aid with PDA detection and guide treatment decisions. We hypothesize that increased shunt exposure could lead to increased risk of kidney injury; specifically, as previous work has demonstrated differences in renal NIRS between neonates with and without a PDA, kidney injury may be identified sooner and lead to more appropriate timing of PDA treatment [78]. Levels of more commonly measured serum biomarkers, including BNP and cardiac troponin, have been used to predict hsPDA versus non-hsPDA versus no PDA, so it is possible that kidney related biomarkers may be able to be used similarly [79]. It is plausible that changes in certain biomarkers may aid determining higher risk populations and timing of ductal therapy. Possible additional research questions are highlighted in Table 3.

**Table 3:** Knowledge gaps and research priorities.

Gap		Research Priority
How does a hemodynamically significant PDA, defined by composite measures indicating moderate to high volume shunting, influence rates of AKI in the NICU?	**→**	Investigate the relationship between PDA shunt volume and short/long term kidney outcomes.
Can kidney dysfunction determine PDA management decisions?	**→**	Determine if new kidney biomarkers can detect nuances in PDA pathology and higher risk populations
Does increased shunt exposure increase rates of AKI? CKD?	**→**	Correlate rates of AKI and CKD with duration of shunt exposure.
Are different PDA treatment methods more protective against CKD development?	**→**	Determine if treating PDAs medically, procedurally, or both influence both short- and long-term kidney outcomes.
Can biomarkers differentiate kidney dysfunction in neonates with hemodynamically significant PDAs?	**→**	Determine patterns in NIRS trends and other biomarkers in neonates with a PDA.
Can biomarkers be used to predict treatment success or failure in neonates with hemodynamically significant PDAs?	**→**	Evaluate how NIRS and biomarkers change with PDA treatment.
Can NIRS be used to stratify infants with hemodynamically significant PDAs at risk of short and long term kidney damage?	**→**	Determine whether a patterned change takes place in NIRS values in neonates with a PDA that varies with AKI stage.
